# Editorial: Interaction between food homologous plants and intestinal microbiota

**DOI:** 10.3389/fmicb.2023.1230234

**Published:** 2023-08-14

**Authors:** Muhammad Fakhar-e-Alam Kulyar, Wen Zou, Sijia Lu, Kyein San Loon, Muhammad Akhtar, Awais Ihsan, Shah Nawaz, Ahmed Ezzat Ahmed, Mohamed Abdelrahman, Yi Wu, Mujahid Iqbal, Kun Li

**Affiliations:** ^1^Institute of Traditional Chinese Veterinary Medicine, College of Veterinary Medicine, Nanjing Agricultural University, Nanjing, China; ^2^College of Veterinary Medicine, Huazhong Agricultural University, Wuhan, China; ^3^Department of Biosciences, COMSATS University Islamabad, Sahiwal Campus, Sahiwal, Pakistan; ^4^Department of Biology, College of Science, King Khalid University, Abha, Saudi Arabia; ^5^Key Laboratory of Agricultural Animal Genetics, Breeding and Reproduction, Huazhong Agricultural University, Wuhan, China; ^6^Animal Production Department, Faculty of Agriculture, Assuit University, Assuit, Egypt; ^7^Department of Pathology, Cholistan University of Veterinary and Animal Sciences (CUVAS), Bahawalpur, Pakistan

**Keywords:** gut microbiota, gut-disease relation, food plants, intestinal barrier, microbiota-based therapeutics

In recent years, experts have recognized the critical role of gut microbiota in maintaining overall health. The microorganisms, including bacteria, fungi, and viruses, living in the intestine are referred to collectively as the intestinal microbiota, which play a significant role in several physiological processes in the digestion and metabolism of numerous food constituents (Altveş et al., 2020). The interaction between food-homologous plants and the digestive tract bacteria is one component of this intricate interplay. Plants are considered food homologous if they have specific bioactive chemicals comparable to those found in food normally. Studies have demonstrated that these compounds, also known as phytochemicals, can modify the composition and activity of the gut microbiota under the influence of several variables, including genetics, food, and lifestyle. Moreover, research has shown that certain phytochemicals found in food homologous plants may perform the function of prebiotics and provide a source of nutrition for healthy gut microbiota (Orloff et al., 2016) (Figure 1).

**Figure 1:**
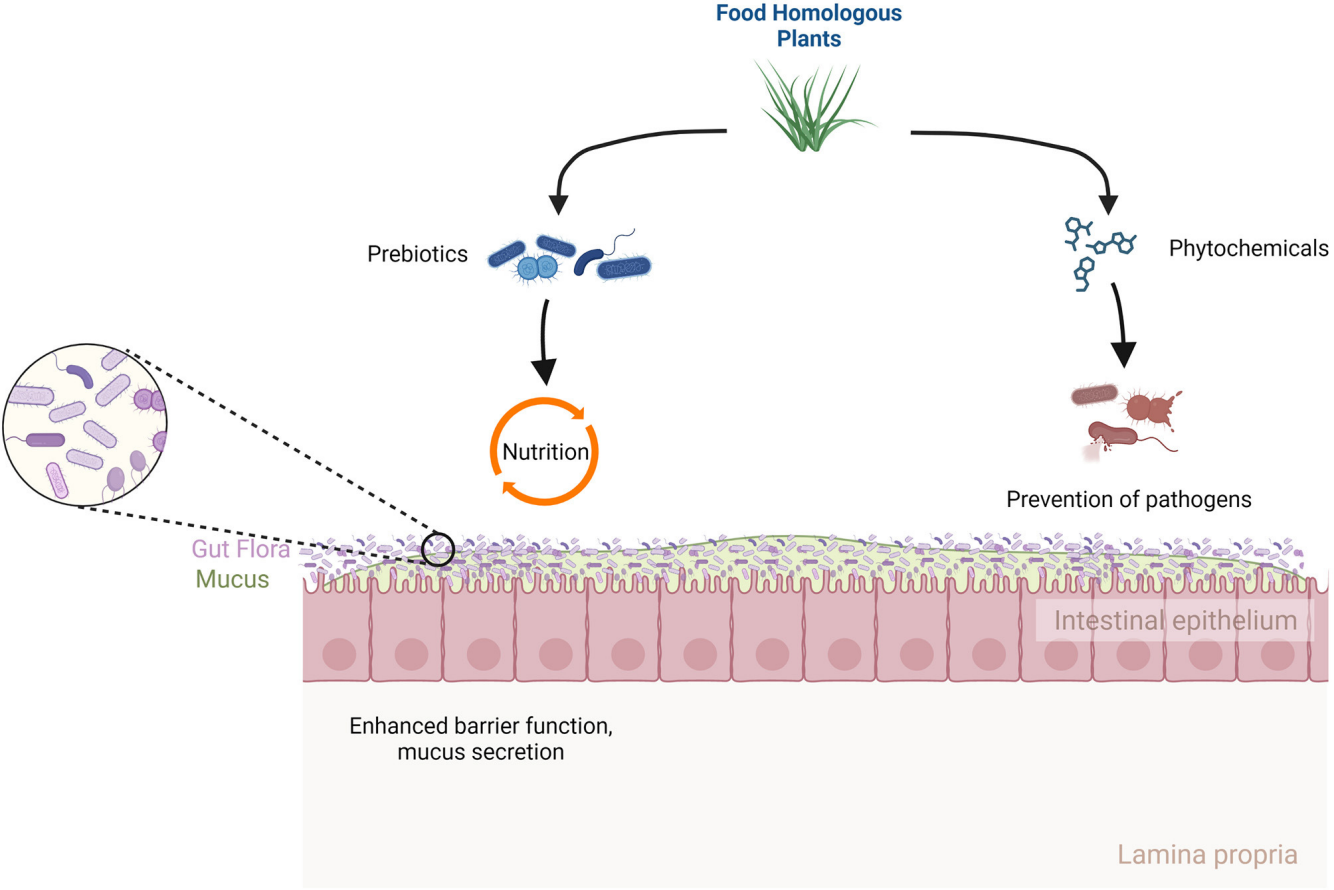
The ways of food homologous plants to prevent pathogens and nutrition to gut flora.

These phytochemicals bypass the digestive tract and end up in the colon, feeding beneficial microbes, such as *Bifidobacteria* and *Lactobacilli*. In addition, it has been shown that food homologous plants exhibit antibacterial characteristics that protect against disease-causing microorganisms. Phytochemicals, i.e., polyphenols and flavonoids, have antimicrobial properties that maintain a healthy gut flora balance and prevent the expansion of hazardous pathogens, which proves a correlation between food homologous plants and their remodeling toward the microbiota of the digestive system. Recent studies have demonstrated that the microbiota in the gut may convert phytochemicals into bioactive molecules that may improve the host's health, e.g., short-chain fatty acids (SCFAs) (Den Besten et al., 2013). The advantages of these SCFAs include enhanced energy metabolism, less inflammation, and better gut barrier integrity, that play an important role in the microbiome of the entire digestive system.

There is a very distinct relationship between food homologous plants and the bacteria in the gut of a host, which might be influenced by various variables, including age, genetics, and other health issues, regardless of the enormous variation between different hosts in the composition of their gut microbiota. The complex link between such plants and the microbiota of the digestive tract significantly affects overall health. Modulating the microbiota in the gut via the ingestion of these plants has the potential to not only maintain a healthy gut environment but also manage or prevent various health issues. Moreover, idling certain phytochemicals and their metabolites (responsible for the observed effects on the gut microbiota) might lead to targeted therapies and personalized dietary recommendations.

As a result of the ongoing expansion of study, several essential subfields of research have recently come to the fore. One of those areas focuses on the effect of food homologous plants on the microbial diversity in the gut. Numerous scientific investigations have shown a correlation between normal and abnormal microbiota in the gut. It is hypothesized that an enormous variety of helpful bacteria in the gut might contribute to better immune function, increased nutritional absorption, and protection against hazardous pathogens. This is because beneficial bacteria can inhibit the growth of harmful pathogens (Wu and Wu, 2012). Therefore, consuming a wide array of such plants has been associated with higher levels of microbial diversity in the gut, which is an interesting finding. Because these plants contain a vast spectrum of phytochemicals, they may supply a wide variety of substrates for the development and metabolism of microorganisms, encouraging a more diversified ecology of microorganisms. In addition, the interaction that takes place in the gut may have an effect on the creation of metabolites that have an impact on health. For example, some bacteria in the gut can transform chemicals derived from plants into metabolites with anti-inflammatory characteristics. These metabolites can potentially assist in regulating the immune system and protect against chronic inflammation that is associated with several disorders, including inflammatory bowel disease, cardiovascular disease, and some forms of cancer (Belkaid and Hand, 2014). Insight into the potential therapeutic uses of food homologous plants may be gained via understanding the unique processes by which these plants affect the metabolic activity of gut microbes. Furthermore, some phytochemicals can affect the metabolism of dietary fats and carbohydrates, which in turn may affect the control of weight and the development of metabolic illnesses such as diabetes and obesity. It has been shown that the microbiota in the gut contributes to the digestion and absorption of nutrients, hence playing an essential part in the metabolic processes being described.

The interaction between food-homologous plants and the microbiota of the digestive system has implications for brain functioning. The gut and the brain can interact through the gut-brain axis. Emerging research shows that abnormalities in the gut microbiota composition, often known as dysbiosis, may contribute to developing mental health issues such as depression and anxiety (Clapp et al., 2017). These illnesses may be prevented by maintaining a healthy balance in the gut microbiota. Additionally, the consumption of such plants has been shown in many studies to benefit the gut microbiota, mental state, and cognitive performance. Moreover, the gut microbiota is thought to generate neurotransmitters and other signaling chemicals that may impact brain function. However, the specific processes that underlie it are still being investigated. The Research Topic “*Interaction between food homologous plants and intestinal microbiota*” comprehensively explores the intricate relationship between traditional Chinese herbal medicines, plant-derived compounds, and seaweed polysaccharides with the gut microbiota. The articles highlight the potential therapeutic applications and underlying mechanisms by which these compounds interact with the gut microbiota to improve health outcomes. Several studies focused on the effects of traditional Chinese herbal medicines on various aspects of health. Danggui Buxue decoction (DBD) exhibited promising effects on type 2 diabetes. DBD treatment improved insulin sensitivity, reduced inflammation, and enhanced microbial diversity in the intestines, suggesting its potential as an antidiabetic and anti-inflammatory agent (Wang et al.). Similarly, Astragalus polysaccharides (APS) showed anti-inflammatory effects with lung injury induced by lipopolysaccharides (LPS). APS improved lung pathology and reduced inflammation, possibly through its modulation of gut microbiota (Ming et al.).

The interaction between plant-derived compounds and gut microbiota was also evident in studies involving animal models. Taxifolin, a natural flavonoid, enhanced semen quality in Duroc boars by modulating gut microbes and blood metabolites. It increases sperm motility and beneficial blood components while reducing harmful bile acids and cholesterol levels (Zhou et al.). Low-nicotine tobacco (LNT) supplementation in rabbits showed promising results on health parameters, altering gut microbiota diversity and metabolites. LNT positively impacted blood status and carcass weight, indicating its potential as a feed additive (Jing et al.).

Other studies delved into the immunomodulatory effects of Chinese medicine compounds. The compound small peptide of Chinese medicine (CSPCM) alleviated cyclophosphamide-induced immunosuppression in mice by modulating gut microbiota and regulating Th17/Treg balance. CSPCM increases immune organ indices, anti-inflammatory cytokines, and beneficial gut bacteria while decreasing immunosuppressive Treg cells and harmful bacteria (Cui et al.). Black Lycium barbarum polysaccharide (BLBP) demonstrated protective effects against lipopolysaccharide (LPS)-induced intestine damage. BLBP attenuated inflammation and improved intestinal morphology, possibly through the NAD(P)H dehydrogenase (quinone) 1/NF-κB pathway and modulation of gut microbiota (Yan et al.). The potential of seaweed polysaccharides as therapeutic agents was also explored. Seaweed polysaccharides alleviated hexavalent chromium-induced gut microbial dysbiosis, demonstrating their ability to restore gut microbiota balance (Mu et al.).

Overall, the articles in this Research Topic collectively emphasize the importance of gut microbiota in mediating the health benefits of traditional Chinese herbal medicines, plant-derived compounds, and seaweed polysaccharides. These studies shed light on novel therapeutic strategies for various health conditions, including diabetes, inflammatory lung injury, immunosuppression, and gut microbial dysbiosis. The findings underscore the significance of understanding the complex interactions between food-homologous plants and gut microbiota for developing innovative and effective therapeutic interventions.

Although studies on food-homologous plants have been conducted, many obstacles still need to be overcome. Determining which particular phytochemicals are responsible for the effects that have been seen is a significant obstacle. It is challenging to isolate and analyze the impact of individual components since many identical plants possess a complex variety of bioactive substances. This makes it tough to examine the effects of individual components. Moreover, the fact that the gut microbiota is uniquely composed presents a hurdle when formulating applicable dietary guidelines.

In conclusion, food homologous plants' interactions with the microbiota of the digestive tract is fascinating. The chemical compounds present in such plants can change the layout and activity of the gut microbiota, which might positively affect health. However, further study is required to understand the underlying processes and locate the particular phytochemicals that are accountable for the impacts that have been reported. This information might pave the way for personalized dietary plans to improve gut and body health.

## Author contributions

MK wrote the first draft of the manuscript. All other authors have made a substantial, direct, and intellectual contribution to the work and approved it for publication.
